# Endodontic emergency patients’ profile and treatment outcome – a prospective cohort study

**DOI:** 10.1186/s12903-024-05338-8

**Published:** 2024-12-26

**Authors:** Sivakami Rethnam Haug, Margrethe Røegh, Inge Fristad

**Affiliations:** Department of Clinical Dentistry Section of Endodontics, The Faculty of Medicine, University of Bergen, Bergen, Norway

**Keywords:** Pain, Diagnosis, Decision making, Pulpotomy, Prognosis, Outcome

## Abstract

**Background:**

Toothache is a debilitating condition, often with mild to excruciating pain, swelling, eating difficulties and insomnia. This study aims to delineate the profiles of patients seeking emergency dental care, focusing on the diagnosis, treatment, and outcomes following non-surgical root canal treatment.

**Methods:**

This prospective cohort study was conducted from 2012 to 2021 at the Section for Endodontics, Department of Clinical Dentistry, University of Bergen, Norway. A total of 281 emergency patient forms were analyzed. Data registered included patient demographics, dental history, chief complaints, medications used, diagnostic results, treatments provided and outcome.

**Results:**

A total of 272 patients (272 teeth) were included in the study. Pain was the predominant complaint (98.5%), where only 57.4% of the patients managed to localize pain to a specific tooth. The mean age of patients was 51.2 years with no significant gender differences. The maxillary right first molar (15.4%) was the most frequent tooth needing treatment. The majority of the patients had experienced pain for three days before they attended the emergency appointment. The most frequently used drug for pain management was paracetamol which was stated to have little effect. Teeth that needed endodontic treatment often had restorations rather than caries. The most frequent diagnoses were pulpitis (26.8%) followed by necrotic pulp (25.4%) and previously root filled teeth (22.8%). Root canal treatment was performed on 60% of the teeth and a success rate of 95% was registered at one-year recall.

**Conclusions:**

There was no singular diagnostic cause leading patients to seek an emergency appointment, highlighting the necessity of a thorough diagnostic procedure. Over the counter pain medications have little effect on alleviating dental pain, often resulting in desperate measures of self-medication. 60% of the teeth needing emergency treatment had previous coronal restorations such as fillings or crowns, indicating that conservative treatment does not appear to fully protect against future pulpal disease. The good prognosis of root canal treatment for teeth with acute symptoms supports recommending dentists to attempt root canal treatment rather than opting for tooth extraction.

**Supplementary Information:**

The online version contains supplementary material available at 10.1186/s12903-024-05338-8.

## Background

Toothache is a debilitating condition that often drives people to seek emergency dental treatment. A study from Denmark indicated that endodontic-related infections were responsible for dental pain in 66% of the patients who sought treatment [1]. If left untreated, infection in a dental pulp can spread to the alveolar bone, soft tissue, muscles and even into the bloodstream. In the United States, approximately 7,000 patients are hospitalized annually due to complications from endodontic infections [2]. In the most severe cases, these infections or resultant septicemia can be fatal [3–5]. Historical records, such as the “Bills of Mortality” from early 17th-century London, show that dental problems were among the top six causes of death at the time [6, 7].

Dental professionals often encounter patients who require emergency treatment that cannot be delayed until a scheduled appointment. Our previous research has identified a psychosocial component to acute dental pain. Stress, whether originating from home or the workplace, can activate the immune system and exacerbate an existing asymptomatic endodontic condition [8]. Patients experiencing acute dental pain have been found to exhibit elevated levels of salivary cortisol — a hormone that increases under stress — along with inflammatory mediators such as interleukin-1β (IL-1β) and IL-6 [8].

In addition, a study by Dahlström in 2016, revealed that general dental practitioners often experience stress, frustration, and a sense of lack of control during endodontic procedures [9]. Needless to say, it may be challenging for a dental practitioner to diagnose and perform emergency treatment within a limited time in situations when a patient turns up with pain and/or swelling [10]. It may also represent a diagnostic challenge that patients with excruciating dental pain often have difficulties localizing pain to a specific tooth. To provide optimal treatment, diagnosis needs to be established following a clinical and radiographic examination. An interim treatment plan should be devised for the acute phase to alleviate the patient’s symptoms, followed by a formulation of a permanent or definitive treatment plan, often including multi-disciplinary and economic considerations. Research regarding emergency treatment in the field of Endodontics is limited, making it imperative to prioritize this area of study. Enhanced understanding and knowledge will ultimately lead to improved treatment options and outcomes for patients.

The objectives for emergency treatment can be divided into short-term and long-term goals. The immediate focus is to alleviate pain, control infection, and prevent systemic complications. The long-term objective is to prevent re-infection in the root canal system and alveolar bone [11]. Research has demonstrated that preserving a tooth through endodontic treatment contributes to a better quality of life compared to extraction [12, 13]. Patients seek dental treatment for a variety of reasons. Within the context of this study, emergency treatment refers to instances where patients have a dental problem that, according to them, cannot wait for a scheduled appointment.

The aim of this study was to establish a comprehensive profile of patients attending emergency dental appointments. This includes documenting signs and symptoms, identification of affected teeth, the diagnostic process, medication used, and the assessment of both emergency and definitive treatment plans. Additionally, the study will examine the outcomes of root canal treatments provided under these emergency conditions.

## Methods

This study is a prospective cohort study conducted from 2012 to 2021. It included all patients who attended an emergency dental appointment at the Section for Endodontics, Department of Clinical Dentistry, Faculty of Medicine, University of Bergen, Norway. The patients were registered using an Emergency Patient form (Appendix 1), designed to record profiles of patients receiving emergency dental care, including their diagnosis and both interim and permanent treatment plans. The Emergency Patient form is available for download (Appendix 1).

Dental students who attended to these patients were responsible for filling out the Emergency Patient form and a quality control was conducted by the clinical instructors. The information from the Emergency Patient forms was anonymized and transferred to an Excel file. Additional information for clarification and treatment outcomes was collected from the patient records. A treatment outcome was considered successful when the patient had no complaints, the tooth had an adequate coronal seal, presented with no tenderness to percussion or palpation, had no deep periodontal pockets, showed no signs of sinus tracts, and demonstrated no periapical radiolucency. A tooth was registered with uncertain treatment outcome when tooth had no clinical symptoms, but where the periapical radiolucency had not diminished in size. A tooth was considered to have failed when the periapical lesion was larger than the preoperative radiograph, presented with sinus tract or deep pockets or was extracted.

### Human ethics and consent to participate

The research protocol was approved by the Regional Committees for Medical and Health Research Ethics West, Norway as a quality assurance study (REK 617936). The need to consent to participate was waivered by the Institutional Review Board and the Regional Committees for Medical and Health Research Ethics West, Norway as this was categorized as a quality assurance study (REK 617936). The study was also approved by the Department of Clinical Dentistry. Throughout this process, all regulations for GDPR, as legally mandated by the EU for all EU and EEA countries, were strictly adhered to. Clinical trial number is not applicable to this study.

#### Inclusion and exclusion criteria

During the period under review, a total of 281 forms were collected from all patients attending an emergency appointment. Patients lacking registration number or cannot be traced to treatment details were excluded. Nine forms missed the patient’s registration number and were consequently excluded from the study. As a result, 272 forms were finally included in the analysis.

### Statistical analyses

Student t test and Mann-Whitney rank sum test were used to determine statistically significant differences between groups. All analyses were performed using Statistical Package for the Social Sciences (SPSS, version 26.0, IBM). A *p* value of 0.05 or less was considered statistically significant.

## Results

**Fig. 1:**
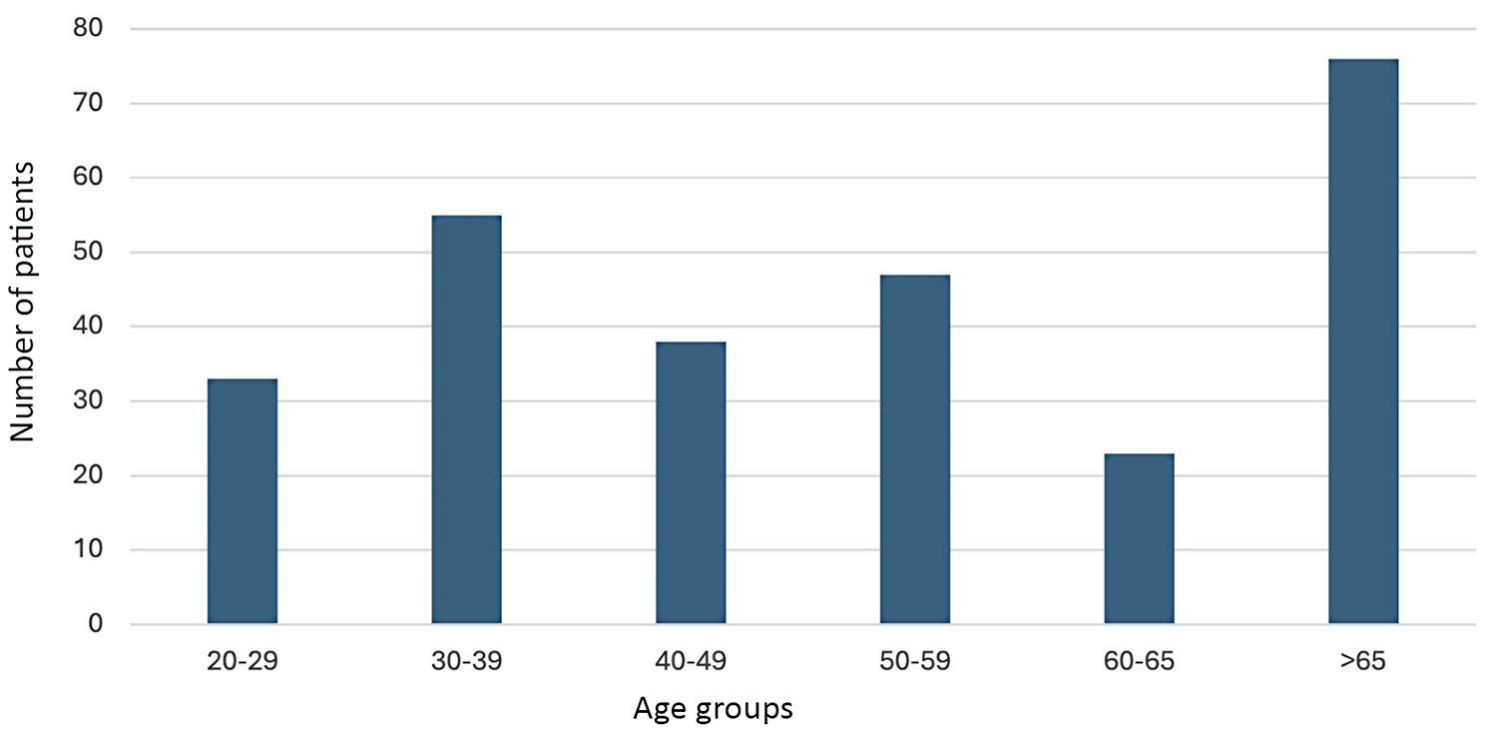
Number of patients (y-axis) attending emergency appointment according to age groups (x-axis)

A total of 272 (143 male and 129 female) patients with 272 teeth was included in the study, showing no significant gender difference. The average age of patients seeking emergency treatment was 51.2 years, ranging from 20 to 90 years. The most frequent age group attending an emergency appointment was patients over 65 years, accounting for 29.9% of cases, although no significant differences between the age groups were observed (Fig. 1).

**Fig. 2:**
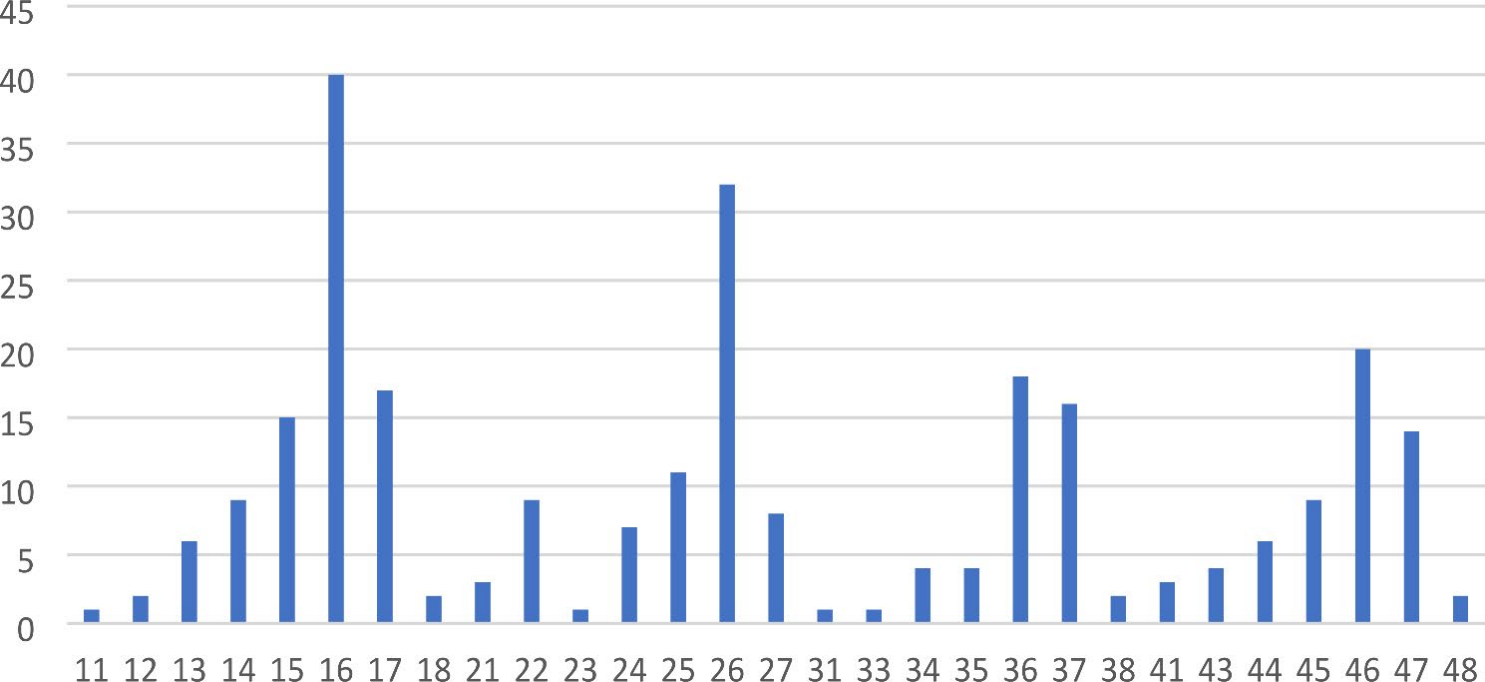
Number of teeth (y-axis) requiring emergency appointment according to FDI tooth numbering system (x-axis)

The tooth most commonly requiring emergency treatment was the maxillary right first molar (15.4%), followed by the maxillary left first molar (11.8%) and the mandibular right (7.4%) and left first molars (6.6%), respectively (Fig. 2). Of all the teeth involved, 61% were located in the maxilla and 39% in the mandible. Teeth that did not present with any emergency problems included the mandibular lateral incisors and the maxillary left third molar.

**Fig. 3:**
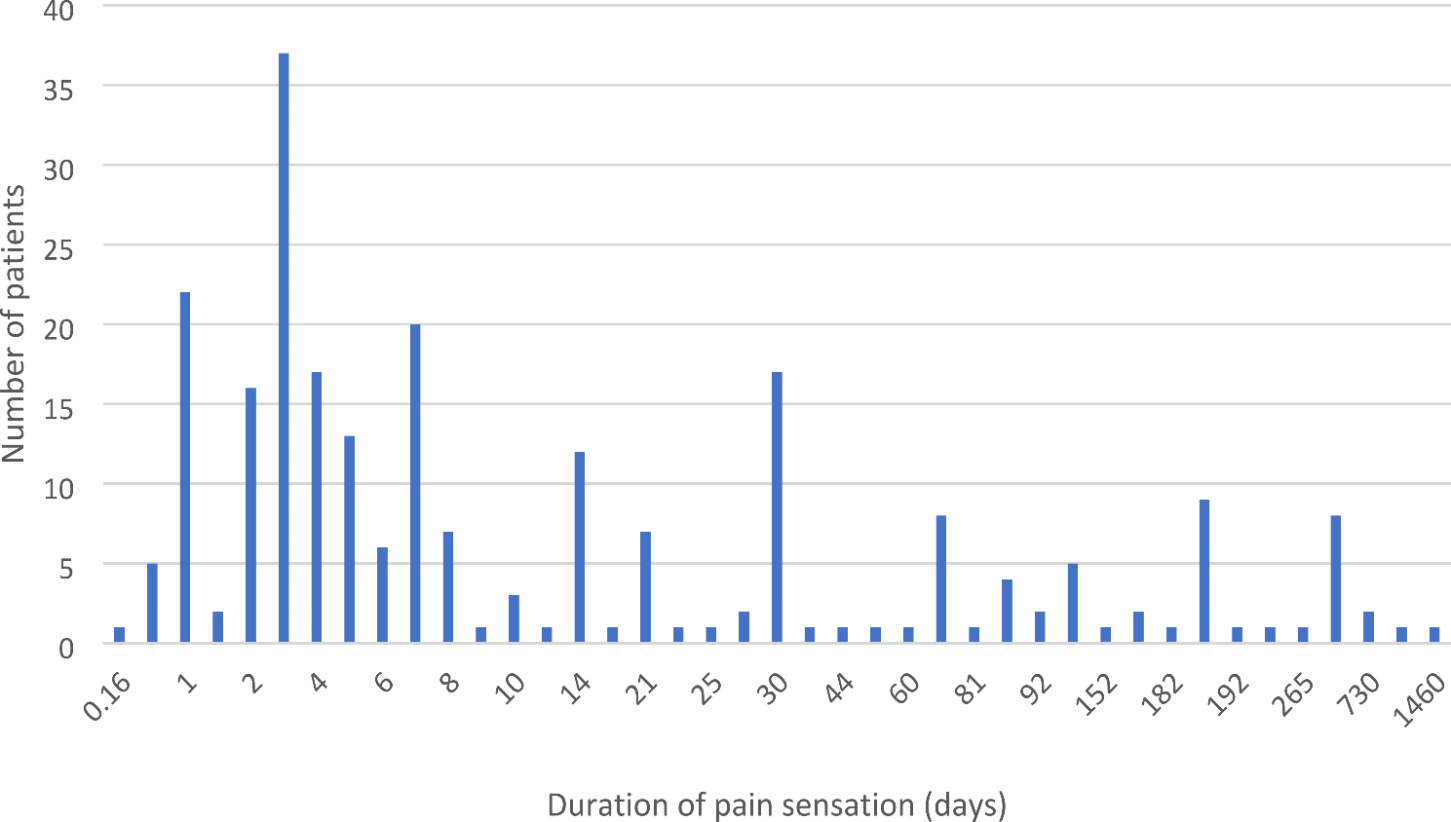
Number of days the patients experienced pain before they attended the emergency appointment

Pain is the most frequent chief complaint by patients (Table 1). Other symptoms included pain on biting or chewing, pain-related sleep disturbances, pain while consuming hot or cold beverages or food, swelling and fever (Table 1). The duration of pain experienced by patients varied widely, ranging from 4 h to 4 years with a mean of 49.1 ± 36.9 days. The most frequent day for seeking emergency appointment was on day 3 (13.6%) following onset of pain. More than half the patients (51.1%) sought emergency appointment within one week, 70.6% of patients took contact for emergency treatment within one month, 14.3% of patients waited for 3 months or longer before seeking help (Fig. 3).

**Table 1:** Symptoms reported by patients as a reason for emergency appointment. Several patients reported more than one symptom

Symptoms			Frequency	Percent (%)
Pain			268	98.5
Pain when biting/chewing			168	61.8
Insomnia (due to pain)			155	57.0
Worsening of pain sensation with hot or cold drinks			110	40.4
Swelling			67	24.6
Fever			19	7.0

A significant portion of the patients, 176 or 64.7%, used one or more types of medications to alleviate pain prior to their emergency appointment (Table 2). Of these patients, 61.4% took one type of medication, 34.7% used two types, and 3.9% combined three different medications. The self-administered medications included over-the-counter analgesics, and prescription drugs such as antibiotics, opioids, among others (Table 2). The most commonly used analgesic was paracetamol, utilized by 61.9% of those who took medications. Patients often resorted to taking combination drugs or stronger painkillers, as over-the-counter medications did not provide sufficient pain relief.

**Table 2:** Overview of medications used by patients for pain relief

Medications	Number of patients	Percentage (%)
**Analgesics**		
Paracetamol	109	39.0
**NSAIDS**		
Ibuprofen	88	32.4
Voltarol (Diclofenac)	1	0.4
Disbril (Aspirin)	1	0.4
Retard	1	0.4
**Antibiotics**		
Apocillin	5	1.8
Amoxicillin	2	0.7
Dalacin	1	0.4
Uspecified	1	0.4
Metronidazol	1	0.4
**Opioides**		
Tramadol	2	0.7
Codaxol (paracetamol with codeine)	1	0.4
Paralgin forte (paracetamol with codeine)	24	8.8
Pinex forte (paracetamol with codeine)	7	2.6
Morphine	1	0.4
Buprenorphine	1	0.4
**Other medications**		
Imovane (Z-drug hypnotics)	1	0.4
Sobril (benzodiazepam)	1	0.4

Only 156 patients, or 57.4%, were able to accurately localize the pain to a specific tooth (Table 3). The majority of these teeth had restorations (61.8%), while only 19.1% presented with carious lesions. The most frequent pulp diagnosis was pulpitis (26.8%), followed by pulp necrosis (25.4%) and previously root filled teeth (22.8%). The remaining patients presented with pain from periodontal disease or problems associated with fractured or missing fillings.

**Table 3:** The patient demographic information. Recurrent caries on teeth with restorations and prosthodontics was registered as caries

Variables	Categories	Frequency *n* (%)
Pain localization	Single tooth 2–5 teeth One quadrant Two quadrants	156 (57.4) 33 (12.1) 79 (29.0) 4 (1.5)
Coronal status	Restoration Prosthodontics Caries	168 (61.8) 55 (20.2) 52 (19.1)
Pulp diagnosis	Pulpitis Pulp necrosis Previously root filled tooth Others	73 (26.8) 69 (25.4) 62 (22.8) 68 (25.0)

The most common emergency treatment plan was pulpotomy (25.4%), followed by medications (20.6%), and root canal debridement for necrotic cases (19.5%) (Table 4). The definitive treatment (permanent treatment) that the patients received after emergency appointment, is presented in Table 5. In total, 163 teeth underwent non-surgical root canal treatment and non-surgical retreatment (103 molars, 40 premolars and 20 anteriors), and 109 patients returned for a one-year follow-up appointment. Of these, 103 teeth (95%) were evaluated to have had a successful outcome, 2% were considered uncertain, and 3% were classified as failures (Table 6). All failures were in the molar group.

**Table 4:** Emergency treatment plan. Since several patients received more than one treatment, the total is more than the patient number in the study

Emergency treatment plan	Number	Percentage (%)
Pulpotomy	69	25.4
Medications/Drugs	56	20.6
Root canal debridement (necrotic pulp)	53	19.5
Restorative treatment	25	9.2
Extraction	25	9.2
Referral to other specialities	22	8.1
Observation	21	7.7
Periodontal therapy	7	2.6
Incision and drainage	6	2.2

**Table 5:** The definitive treatment patients received. Since several patients received more than one treatment, the total is more than the patient number in the study

Definitive treatment	Number	Percentage (%)
Non-surgical root canal treatment	156	57,4
Extraction	39	14.3
No treatment	22	8.1
Patient did not return	18	6.6
Restorative or prosthodontic treatment	14	5.1
Periodontal therapy	9	3.3
Non-surgical retreatment	7	2.6
Surgical retreatment (hemi-section, root amputation or endodontic surgery)	7	2.6

**Table 6:** Treatment outcome for teeth that received root canal treatment

Outcome	Number	Percentage (%)
Successful	103	95
Uncertain	2	2
Failure	4	3
Total	109	100

## Discussion

The main findings of this study indicated that patients often delayed seeking an emergency dental appointment until experiencing significant pain, which served as the primary motivating factor. Restorative treatments, such as fillings or crowns, do not fully protect against pulpal disease. A notable proportion of cases requiring emergency treatment involved previously root-filled teeth. Additionally, patients across all age groups experienced pain, with first molars being the most commonly problematic teeth.

The dental pulp, when inflamed, is capable of eliciting extreme pain, reaching the highest scores on pain rating scales [14]. On the other hand, a necrotic dental pulp is theoretically incapable of giving rise to pain sensation. Interestingly, this study found nearly equal incidences of pulpitis and pulp necrosis. As the disease progresses within the dental pulp, a mixture of vital tissue and necrotic remnants may be present, leading to diagnoses such as partial necrosis or pulp necrobiosis, where the remaining vital pulp tissue can still cause pain [8]. Additionally, a necrotic pulp can lead to symptomatic apical periodontitis, manifesting as pain during biting and chewing [15].

Previous emergency treatment modalities [7], have been based on removal of vital pulp tissue and necrotic tissue remnants from the root canal system. Therefore, an unexpected finding in this study was the complications associated with previously root filled teeth. A previously root filled tooth can fail due to problems associated with coronal restorations, the root filling itself, root fractures or periodontal problems. With more patients choosing to retain their natural teeth, a rise in non-surgical retreatments of previously root-filled teeth is expected [16]. However, surgical retreatment was an indication in 2.6% of patients as a definitive treatment plan. This option was chosen in cases where the root canals were not accessible for non-surgical retreatment or when extraradicular infection was suspected.

The quality of root canal treatment is imperative to a successful treatment outcome. Therefore, root canal treatments should adhere to a high standard of care during primary procedures. Besides the mentioned high standard of endodontic treatment, the survival of an endodontically treated tooth seems to be affected substantially by modern post-endodontic restorations in the long-term [17]. A study from Norway concerning endodontic treatment needs in general dental practice revealed that 11.6% of cases were related to previously root-filled teeth, a finding that coincides with this study where 9.5% of the planned permanent treatments involved surgical and non-surgical retreatments [18]. Furthermore, 8.1% of patients were referred to other specialists for prosthodontic treatments or surgical management of patient.

The most frequent teeth requiring emergency dental treatment were the first molars. This finding is perhaps expected, as these teeth erupt around the age of six and have been in function for the longest period. On the other hand, the incisors in the lower jaw, which erupt at the same time, exhibit the lowest frequency of emergency cases. This discrepancy suggests that multiple factors influence the frequent occurrence of issues with molars. Possible explanations include differences in anatomical complexity, missed and untreated root canals in previously root filled teeth, and their position within the oral cavity. A study investigating caries prevalence in permanent dentition noted that teeth with deep and complex fissures were at the highest risk [19]. Similarly, it was found that teeth in the upper jaw were more frequently affected by caries than those in the lower jaw, agreeing with the findings of this study and our previous research [20, 21].

No gender differences were observed among patients seeking emergency care, which is consistent with findings from a study by Haug and Marthinussen, indicating that women and men experience similar levels of pain related to pulpal and periapical inflammation [8]. Additionally, no specific age group was disproportionately affected, as the age range of patients stretched from 20 to 90 years, suggesting that individuals of all ages experience dental pain and require emergency dental care.

A study on dental emergencies in Finland pointed out that carious lesions and its sequelae were the primary causation [22]. However, in this study, most of the affected teeth had undergone some form of previous restorative treatment, with many patients displaying fillings. This highlights that patients who make regular dental visits, unfortunately, may face emergency situations and that restorative treatment, such as fillings or crowns, may cause pulpal disease as an adverse event in the long-term. Pulpal injury may occur as a result of caries, repeated restorative treatment, extensive removal of enamel protecting the dentin, heat generated during preparation, lack of water cooling, excessive drying of denuded dentine, or marginal defects with persistent microleakage [23]. However, meticulous restorative treatment of a damaged tooth is essential to avoid an ongoing process of tooth destruction due to secondary caries or fracture, resulting in a non-restorability.

In this study, the most commonly used medication to alleviate pain was paracetamol, followed by ibuprofen, a finding similar to our previous study [8]. A surprising observation was the wide variety of drugs used by patients for pain relief, including opioids and antibiotics. This diversity in self-medication underscores the need for more effective analgesics to alleviate dental pain. Our previous study reported that 83% of patients reported insomnia despite ingesting painkillers [8]. Additionally, the desperation to relieve pain emphasizes the substantial impact of dental pain on oral health-related quality of life. Furthermore, a study on individuals suffering from insomnia demonstrated that sleep disturbances significantly deteriorate their quality of life [24].

Living with pain significantly affects individuals’ ability to function well both physically and mentally [25, 26]. Most patients cited pain sensation as their primary reason for seeking emergency treatment, with about 70% experiencing pain severe enough to necessitate medication. Consequently, the goals of emergency dental treatment are to relieve pain, control or prevent infection and enhance patients’ well-being and quality of life.

Despite the fact that root canal treatment was performed on patients with preoperative pain and other symptoms, the treatment outcome was largely successful where a success rate of 95% was registered at a one-year recall. Previously, high endodontic success has been reported in follow-up studies at university clinics and private specialist clinics, in contrast to what is found in epidemiological studies [27–30]. Overall, there is evidence that quality of treatment is important for the outcome of endodontics, and that preoperative pain does not affect the long-term outcome as long as the infection is controlled [28, 31]. A favorable prognosis for root canal treatment and non-surgical retreatment of teeth presenting for emergency appointment provides grounds for recommending dentists to perform non-surgical root canal treatment and retreatment rather than extraction during the clinical decision-making phase. Although this study did not specifically examine the number of treatment visits required to complete a root canal treatment, our previous research indicated that, on average, two to three appointments are needed. Molars and cases classified as highly difficult according to the American Association of Endodontists case difficulty assessment form typically require significantly more treatment visits [32]. It is imperative that this information is communicated to the patient as part of the clinical decision-making process.

One of the limitations of this study is that the undergraduate clinic treats only adult patients, and as a result, children and dental trauma patients are not represented. Although the patient cohort at the Department of Clinical Dentistry may not be representative of the general population, the patients attending regular dental treatment at educational institutions can be comparable to a large private practice consisting of general practitioners and specialists. Future studies should focus on educating patients about when to seek dental treatment and on discovery of better medications that can alleviate dental pain.

## Conclusions

There was no singular diagnostic cause leading patients to seek an emergency appointment, highlighting the necessity of a thorough diagnostic procedure. Over the counter pain medications have little effect on alleviating dental pain, often resulting in desperate measures of self-medication. 60% of the teeth needing emergency treatment had previous coronal restorations such as fillings or crowns, indicating that conservative treatment does not appear to fully protect against future pulpal disease. The good prognosis of root canal treatment for teeth with acute symptoms supports recommending dentists to attempt root canal treatment rather than opting for tooth extraction.

## Electronic Supplementary Material

Below is the link to the electronic supplementary material.

Supplementary Material 1

## Data Availability

The datasets used and/or analysed during the current study are available from the corresponding author on reasonable request.
