# Supplementary material for: Endodontic emergency patients’ profile and treatment outcome – a prospective cohort study
Source: BMC Oral Health. 2024 Dec 26;24:1557. doi: 10.1186/s12903-024-05338-8 (PMC11674510; doi:10.1186/s12903-024-05338-8)
Supplement: Supplementary file 1 — Supplementary Material 1 [file 12903_2024_5338_MOESM1_ESM.pdf]

UNIVERSITETET I BERGEN
Odontologisk Universitetsklinikk

Emergency Patient Form

1. How long have you experienced pain? _________ days _________months

2. Evaluate pain using a visual analogue scale (VAS), where 0 corresponds to no pain and 10 corresponds to the worst imaginable pain.

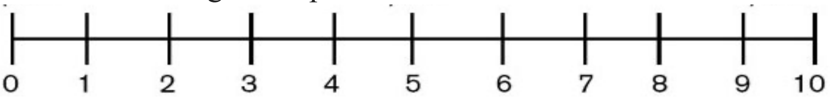

3. Which tooth or region is the pain from? Tooth # ________ Region _________

4. Are you taking (or have taken) any medication for the tooth problem?
☐ Yes . If yes, what type?_______________ ☐ No

5. Can you sleep undisturbed (by the dental problem) at night)? ☐ Yes ☐ No

6. Does the pain get worse after consuming cold or hot food/drink, e.g. ice cream/coffee??
☐ Yes ☐ No

7. Can you bite/chew on this tooth without discomfort? ☐ Yes ☐ No

8. Are you noticing any signs of fever? ☐ Yes ☐ No

9. Are you experiencing any swelling? ☐ Yes ☐ No

10. How does the tooth appear clinically? ☐ Caries ☐ Restoration ☐ Crown ☐ Others

11. How does the tooth appear radiographically?
Crown: ☐ Deep caries ☐ Large restoration near pulp ☐ Crown ☐ Others
Periapical: ☐ Normal periapex ☐ Widening of apical periodontium ☐ Radiolucency ☐ Others

12. Clinical and vitality tests of tooth and adjacent tooth:

Tooth	Tooth #	Percussion	Palpation	EPT	Cold test	Heat test	Perio pockets(mm)
Control							
Test							

13. Diagnosis

Pulp
☐ Pulpitis
☐ Pulp necrosis
☐ Previously rootfilled tooth
☐ Previously pulpotomized tooth
☐ Others: ____________

Periapical
☐ Acute apical periodontits
☐ Chronic apical periodontits
☐ Periapical abscess with sinus tract
☐ Periapical abscess without sinus tract

14. Emergency treatment plan
☐ Pulpotomy (pulpitis) ☐ Root canal debridement (necrotic pulp)
☐ Incision and drainage ☐ Medication (antibiotics, analgesics etc.)
☐ Others: ____________

15. Permanent treatment plan
☐ Root canal treatment ☐ Surgical retreatment ☐Non-surgical retreatment
☐ Extraction ☐ Others: _____________
